# *The African Journal of Laboratory Medicine* – Advancing Laboratory Medicine and Science in Africa

**DOI:** 10.4102/ajlm.v1i1.86

**Published:** 2012-11-15

Laboratory medicine has evolved rapidly on the African continent. The growth of state-of-the art research institutions and public health agencies has led to ground-breaking research, cross-cutting management techniques, and both formal and informal policy statements. Disease-specific and regional laboratory networks have been critical in ensuring that information and practices are shared between neighboring countries. Networks of Excellence have formed in each of the four regions of Sub-Saharan Africa in order to support research infrastructure and strengthen South-South collaboration. In Northern Africa, well-established professional societies, such as the Egyptian Society of Laboratory Medicine and the Moroccan Society of Clinical Chemistry and Laboratory Medicine, play critical roles in the transformation of the laboratory profession. Additionally, laboratory-strengthening activities have been supported by various established organisations, including the South African National Accreditation System (SANAS).

From this fertile intellectual ground have come some of the brightest scientists in the world. Africans on the continent and in the diaspora are leading the way to find preventive measures, tests, and cures for both infectious and non-communicable diseases. Never before have partnerships for global health existed as they do in Africa today, with funding, research, and implementing partners working hand-in-hand with national governments and laboratory systems. International agencies, research institutes, commercial companies, and governmental and regional bodies are joining forces with a common goal: strengthening laboratories and improving health on the continent. Africa has been the catalyst for the development of revolutionary new molecular approaches to address the high burden of infectious diseases, and technologies that can be used closer to the site of patient management. Innovative information tools are being used to support data management, instrument performance, and timely distribution of laboratory results. This is a momentous time in history for laboratory medicine and science in Africa.

The landscape of public health is changing throughout Africa. New infectious diseases are emerging, and as people live longer, non-communicable diseases such as cancer and diabetes are becoming increasingly important, fuelled in this region by high rates of chronic inflammation from diseases such as TB and HIV. As the next tidal wave of challenges for global health rolls in, we must ensure that laboratories are prepared to meet the diagnostic challenges that they will be faced with in the future.

The African Society for Laboratory Medicine (ASLM) was officially launched in March 2011 as an umbrella organisation for African laboratory personnel. ASLM takes an aggressive, forward-looking approach to advancing laboratory services by fostering new research; providing opportunities for mentorship; and building capacity to develop and sustain laboratory practice, science, and policy for better programme implementation and outcomes. The Society’s newly unveiled strategic vision, *ASLM2020*, has set aggressive targets to be achieved by the year 2020: enhancing workforce development by supporting the training and certification of 30 000 laboratory staff; strengthening 25 national and regional regulatory bodies; and enrolling 2500 laboratories in the WHO-AFRO Stepwise Laboratory Improvement Process Towards Accreditation, with at least 250 laboratories achieving accreditation by international standards.

The efforts of ASLM and its partners are beginning to paay dividends, as governments at the highest levels are focusing greater attention on laboratories throughout Africa. Increased recognition of the importance of quality laboratory services and heightened governmental commitment to supporting laboratories are visible throughout the continent. For example, just last month in his State of the Nation Address, President Khama of Botswana lauded the international accreditation of four health laboratories and redoubled his commitment to laboratory accreditation throughout the country.

The expansion of laboratory-related research, management, and policy discussion has led to a critical need for effective and timely communication that advances all aspects of laboratory science on the continent. The *African Journal of Laboratory Medicine*, ASLM’s official scientific journal, is the first all-inclusive peer-reviewed journal specifically designed to cover all laboratory-related topics, with a focus on implications for the African continent. The journal balances cutting-edge basic laboratory science with vital discussions of policy debates, whilst highlighting success stories in which laboratory interventions have made a critical contribution to healthcare programs. The journal focuses on the role of the laboratory and its professionals in the clinical and public health sectors, and supports a data-driven approach to translating research in practice and policy.

At this historical crossroad, the *African Journal of Laboratory Medicine* is well-positioned to provide a critical forum for discussion that advances science and informs policy and programmatic recommendations. We hope that you find this inaugural edition of the *African Journal of Laboratory Medicine* enlightening, inspirational, and thought-provoking.

**The Editors**

